# Computational biology for ageing

**DOI:** 10.1098/rstb.2010.0286

**Published:** 2011-01-12

**Authors:** Daniela Wieser, Irene Papatheodorou, Matthias Ziehm, Janet M. Thornton

**Affiliations:** 1 EMBL–European Bioinformatics Institute, Wellcome Trust Genome Campus, Hinxton, Cambridge CB10 1SD, UK; 2 Institute of Healthy Ageing, UCL, Darwin Building, Gower Street, London WC1E 6BT, UK

**Keywords:** ageing, bioinformatics, computational biology, data analysis, lifespan, gene expression

## Abstract

High-throughput genomic and proteomic technologies have generated a wealth of publicly available data on ageing. Easy access to these data, and their computational analysis, is of great importance in order to pinpoint the causes and effects of ageing. Here, we provide a description of the existing databases and computational tools on ageing that are available for researchers. We also describe the computational approaches to data interpretation in the field of ageing including gene expression, comparative and pathway analyses, and highlight the challenges for future developments. We review recent biological insights gained from applying bioinformatics methods to analyse and interpret ageing data in different organisms, tissues and conditions.

## 1. Introduction

**Figure 1.**
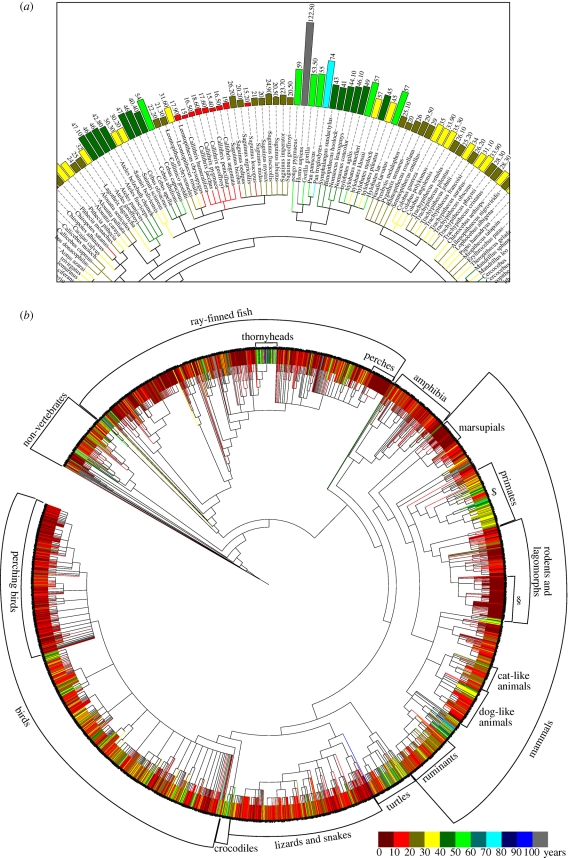
Bioinformatics analysis of phylogenetic clustering of longevity records. The figure was created with the help of the iTOL Webservice [8] using longevity data from AnAge [1] and phylogeny data from NCBI Taxonomy [9]. Similar maximal lifespans can be found in phylogenetically distant species. Within the large variety of maximal lifespans in evolutionary clades, clusters of similar maximal lifespan can be observed. This hints to a strong genetic influence on ageing. (*a*) Excerpt from a primate subtree showing human and closely related primates as well as the *Callitrichinae* family of the New World monkeys. (*b*) Phylogenetic tree of all maximal lifespan data from the AnAge database. $ indicates *Callitrichinae* (a family of New World monkeys), § indicates mouse-like animals.

Ageing is characterized by a progressive decline in organismal fitness that occurs with increasing age, ultimately ending in death. The speed at which this deterioration of physiological function happens, and thus the maximum lifespan, differs for even closely related species—for example, mice live to 3.5 years, Damara mole rats to 15 years and naked mole rats to 28 years (AnAge database [1]). While it has been suggested that exposure to oxidative stress at an early age may provide some protection for the naked mole rat against free radicals at later stages in life [2], the exact mechanisms that lead to such variation in lifespans are still unclear. According to AnAge, some of the longest known living species on earth are the ocean quahog, which lives up to 400 years, and the red sea urchin, which lives up to 200 years. These two species show negligible senescence, i.e. they show neither a change in survival probability with age nor physiological changes such as reduction in reproductive capacity, and nor is there evidence of cellular changes leading to reduced function with age in these animals. The average life expectancy of humans in 2009 in the UK was 79.4 years, while the maximum human lifespan recorded currently is 122.5 years. Figure 1*a* shows that the lifespan of *Homo sapiens* is unusually long among mammals. Figure 1*b* shows a summary of maximal lifespans of evolutionarily distant species. Some groups, such as perching birds, have remarkably homogeneous lifespans while others, including turtles, show wide variation.

While it is unclear exactly what mechanisms drive ageing, it is certain that genes play a role. To date several hundred genes have been identified that either speed up or slow ageing if manipulated in model organisms [1]. An example is the gene *daf-2*, which encodes an insulin-receptor homologue in the nematode worm *Caenorhabditis elegans*. In their seminal work on lifespan extension, Kenyon *et al.* [3] have shown that worm mutants for *daf-2* live twice as long as wild-type worms. Besides such gene manipulations, dietary restriction (DR), i.e. a reduction in food intake that falls short of malnutrition, has been shown to delay ageing [4], whilst manipulation or modification of the endocrine system has been similarly related to the ageing process. For instance, it is well established that the insulin/insulin-like growth factor signalling (IIS) pathway is central to the regulation of lifespan in various organisms [5]. Experimental efforts have been made to investigate such modifications to extend lifespan in laboratory animals including the mouse, fruitfly, worm and yeast, with the aim of extrapolating results obtained from these organisms to humans. This has led to a wealth of publicly available data on ageing, often based on high-throughput genomic and proteomic technologies. These are available through general databases [6,7] and databases specifically developed for ageing [1]. Easy access to these data, and their computational analysis, is of great importance for pinpointing the causes and effects of ageing. For example, identification of related experiments for comparative analyses across different species or within species is important to establish evolutionary conservation of a particular finding.

In this review, we provide a description of the existing databases and computational tools of ageing that are available for researchers. We also describe approaches to data interpretation in the field of ageing, including studies of gene expression and comparative and pathway analyses. We review recent biological insights, gained from applying bioinformatics methods in our laboratory and others, into how to analyse and interpret data on ageing in different organisms, tissues and conditions.

## 2. Resources, databases and tools

**Figure 2.**
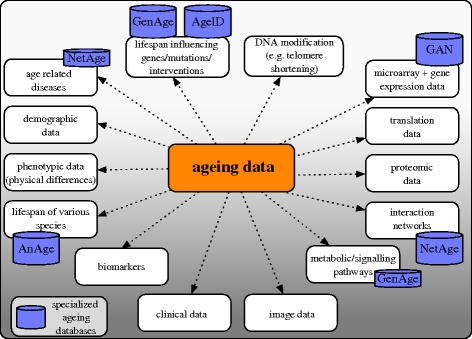
Ageing data and associated databases. Example data types that are linked to ageing are shown. Ageing data can be accessed through general or specialized databases, literature research or on the World Wide Web. Major ageing databases available at the time of writing are indicated in the figure. Examples of general databases include the ArrayExpress, GEO, TRANSFAC, IntAct and Reactome databases.

**Figure 3.**
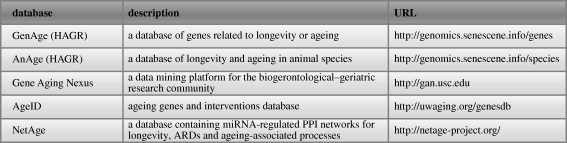
Databases currently available for searching and/or downloading data related to ageing (April 2010).

One challenge in ageing research is to bring together the fragmented data provided by scientists but stored in various locations around the world. These data come in a large variety of types originating from studies in, among others, genomics, transcriptomics, proteomics and metabolomics. Examples for such data types linked to ageing are shown in figure 2 and include gene expression, image or interaction data. Publicly available ageing databases that aim to collect and disseminate the data are shown in figures 2 and 3.

One example is the Human Ageing Genomic Resources (HAGR) [1] database that consists of two subsections, GenAge and AnAge. The former collects genes associated with changes in the ageing phenotype or longevity in model organisms and, at the time of writing (build 14), provided 789 such genes as well as 261 human orthologues for these genes. GenAge secondly identifies genes critical in pathways previously related to ageing, whilst a third dataset in this database contains a list of human genes analysed for their possible association with human longevity. Both positive and negative results from longevity association studies in human are included, with a total of 258 longevity associated studies being available at the time of writing. The GenAge database can be downloaded as tab-delimited file or as an Extensible Mark-up Language (XML) file.

AnAge, the second centrepiece of HAGR, is a database on phenotypes of ageing in animals featuring over 4000 species. Longevity records, developmental and reproductive traits, taxonomic information, basic metabolic characteristics and key observations related to ageing are provided by this database. As an example, the AnAge database was used to create figure 1 with the help of the iTOL webservice [8] using phylogeny data from the National Center for Biotechnology Information (NCBI) Taxonomy [9]. This demonstrates the usefulness of this database for comparative analysis when combined with appropriate bioinformatics tools. HAGR also provides a Perl toolkit named ARCT (Ageing Research Computational Tool) to assist data analysis.

Another web resource for researchers in the field of ageing is the Gene Aging Nexus (GAN) [10] that brings together microarray gene expression data from human, rat, mouse, fruitfly, worm and yeast studies. At the time of writing, 86 datasets comprising 1283 microarray experiments had been provided, while additional datasets can be uploaded by the user. GAN is equipped with a set of tools that enable the user to analyse, query and visualize the ageing-related genomic data of the microarray datasets across different platforms and species.

More recently, the NetAge database [11] became available. It provides access to gene, protein and miRNA interaction networks for yeast, worm, fly and human data, where available. For humans, the data are also linked to age-related diseases. The interaction networks therein are interlinked with the HAGR database.

Various smaller or more specialized ageing databases and tools also exist. For example, the ageing genes and interventions database (AGEID) contains experimental results related to ageing [12] and catalogues published experiments where lifespan has been measured in various organisms. Similar to the AnAge database, AGEID reports contain the organism and strain background in which the particular experiment was performed, the type of genetic or environmental perturbation, the effect on lifespan, a description of the gene function and its role in longevity and protein homologues, together with references. The same research group recently published a software tool Yeast Outgrowth Data Analyzer [13], a program that analyses population survival of yeast cells measured by optical density over time.

Numerous secondary databases and tools that are not directly related to ageing, but nevertheless greatly facilitate the analysis of ageing-related data, are also available. These include the Gene Ontology (GO) [14] which provides exhaustive functional annotation for many genes. The ArrayExpress Archive [6] and associated Gene Expression Atlas as well as the Gene Expression Omnibus (GEO) [7] allow querying and downloading of data related to functional genomics experiments, many of which have some association with ageing. For instance, data produced by the AgeMap project [15] that investigated gene-expression profiles from 16 mouse tissues at various time points can be retrieved from ArrayExpress and GEO. These generic resources ensure long-term access to data, while specialized ageing databases are more likely to be discontinued when a project is completed. The TRANSFAC database [16] provides data on transcription factors, their experimentally proven binding sites and regulated genes. Bioconductor [17] is an open source software project for the analysis and comprehension of genomic data. Clover [18] and Expression Analysis Systematic Explorer (EASE) [19] are programs for identifying functional sites in DNA sequences and for investigating gene lists for functional over-representation of biological terms. The latter three software tools, and many others, have been used extensively to analyse and interpret ageing data.

These databases and tools help to bring together and analyse the deluge of data available on ageing. Nevertheless, these data are still highly fragmented and integration remains a challenging task. To pinpoint exactly which mechanisms are involved in longevity-assurance and ageing, it is critical to provide systems that allow researchers easy access to data from disparate sources, platforms and technologies. Some of the outcomes of applying and using these tools are described in the remainder of the review.

## 3. Data interpretation

### (a) Gene-expression analysis

Analysis of gene-expression data has become an indispensable tool in biological research and has been used extensively in investigations of ageing [15,20–23]. Most available gene-expression data on ageing originate from microarrays, although these are expected to be superseded soon by next-generation sequencing, such as RNA-Seq. In microarray experiments, RNA of at least two different conditions, such as normal and disease or different ages, is hybridized on microarrays. This experimental data collection is followed by bioinformatics analysis detailed elsewhere (ch. 2, 4, 12–15 in Gentleman *et al.* [24]). Briefly, the measured data are first background corrected to remove signals from unspecific binding and other artefacts. This is followed by within- and between-array normalization to compensate for different overall intensity levels caused, for example, by variations in the microarray production. The normalized microarray data are then analysed to determine differential gene expression. Numerous methods have been developed for this analysis, implemented in various software packages. One commonly used approach is linear model fitting followed by empirical Bayes analysis, as provided for example by the R package limma [25]. The software returns a list of genes deemed to be differentially expressed together with multiple statistical measurements of significance for each gene, including *t*-statistics and *p*-values.

An example of differential expression analysis in ageing is the comparison of Ames dwarf mice *Prop*1^*df/df*^ versus *Prop*1^+/+^ and Little mice *Ghrhr*^*lit/lit*^ versus *Ghrhr*^+/*lit*^ [26]. In both cases, the mutants show delayed ageing with significantly increased lifespan (50% and 25%, respectively). The authors found 1125 and 1152 differentially expressed genes in these mutants, respectively, using analysis of variance (ANOVA) with a *p*-value ≤0.0001. A total of 552 of these genes overlapped. This study was also the basis of a cross-species comparison described below.

Once the list of differentially expressed genes has been determined, it can be analysed to extract additional knowledge. One popular tool for this is enrichment analysis, which has been implemented in various tools (e.g. Catmap [27]) and online services (e.g. DAVID [28]). The goal here is to determine whether the up- or downregulated genes are enriched in functional categories, such as disease-associated genes or GO terms. Examples where functional enrichment analysis has been applied include the study in mice mentioned above [26], which found genes involved in metabolism and mitochondrial electron transport to be the most affected in the long-lived mutants. Other examples [22,29,30] using functional enrichment in comparative studies are detailed below. Although analysis of mRNA profiles can identify over-represented gene categories, they may be affected by lack of annotations for some biological processes, but more importantly they can be an experimental dead-end. There is a need for new tools not only to identify relevant biological networks, but also to prioritize genes which would be predicted to have the largest impact on phenotype, and represent good targets for mutagenesis studies. Such tools will need to model pathways and networks and predict with better accuracy the probable consequences of changing a given gene within the network.

Another follow-up analysis often applied after obtaining a gene list is motif analysis, where the goal is to find shared motifs across co-expressed genes, which could indicate a direct co-regulation by the same transcription factor. There are two general ways to address this: *ab initio* motif discovery (e.g. NestedMICA [31]) and library-based motif discovery (e.g. Clover [18]). The big advantage of *ab initio* methods is that they can discover novel motifs. However, motifs have to be strongly defined and/or over-represented in order to be detected, and connecting the motif to a transcription factor can be difficult. In contrast, library-based methods depend on pre-compiled sets of motifs and detect whether these are significantly over- or under-represented in the sequences. Pre-compiled libraries of motifs are offered in open-source databases, e.g. Jasper [32] as well as commercial databases, e.g. TRANSFAC [16]. The methods then use the motifs of the library to find matches in the sequences. Two general advantages of library-based methods are that they can easily include annotation of which motif is bound by which transcription factors, and they seem to be more sensitive in detecting weak motifs. This type of analysis has been used, for example, in the study by Cheng *et al.* [33], which found a number of enriched motifs for three long-lived mutants (*sch9Δ*, *ras2Δ* and *tor1Δ*) compared with wild-type yeast. Among the transcription factors whose motifs were enriched are *Fhl1*, which regulates the transcription of ribosomal protein genes, and *Msn2*/*Msn4*, and *Gis1*, which regulate the expression of many stress-response genes.

Studies that investigate the relationships between genome and transcriptome also benefit from differential expression analysis. In a study of human kidney, the authors found 630 genes that change expression with age [34]. Subsequent analysis of these age-regulated genes found that 101 genes contained expression-associated single nucleotide polymorphisms (SNPs). The advantage of using such a two-stage approach for detecting SNPs is that it reduces the number of genes in the search space and thus the number of statistical tests required to test for SNP association.

Differential expression data can also be used to find correlations between the expression of genes across different experiments. Southworth *et al.* [35] followed such an approach to identify modules of genes whose differential expression is correlated, implying that these genes are being co-regulated. They constructed gene co-expression networks from a collection of microarray profiling experiments on different tissues in mice. The edges of these networks represent the weights of the correlation. They functionally characterized these modules by identifying their enrichment with GO categories, compared them between young and old mice and found a declining correlation trend with age. Finally, using the information on the promoter motifs of these genes, they were able to identify computationally that they are targets of the NF-*κ*B transcription factor.

### (b) Proteomics and metabolomics

While gene-expression analysis using microarrays or next-generation sequencing examines the RNA expression levels, proteomics aims to profile proteins. Different proteomic techniques aim at various aspects, such as protein concentrations, modifications and complexation. There are relatively few proteomic studies directly targeting questions of ageing research compared with gene-expression studies. However, the number of proteomic studies on ageing are increasing (see [36,37], for reviews). An example is the work by Jones *et al.* [38] who investigated protein changes in long-lived *daf-2* mutants, the insulin-receptor homologue in *C. elegans*. The protein changes observed could be related to cellular maintenance and detoxification processes, confirming the outcome of studies using transcriptional analyses. Metabolomics, i.e. the examination of all metabolites in a cell or a tissue in a high-throughput way, has been used to a limited extent in ageing research, too. Furthermore, the impact of metabolite concentrations of, for example, glucose or oxygen species seem to be important in ageing (see also §5*b*). Each of these -omic techniques requires specific computational methods. Combining the results of the different techniques is a general challenge in biological research which needs to be addressed in order to get a more complete understanding. For example, a comparison of long-lived mutants of *daf-2* profiled by microarrays [39], proteomics [40] and metabolomics [41] would be an interesting study in ageing research.

### (c) Pathway analysis

One of the main challenges in interpreting gene-expression data is trying to understand the biological consequences of gene-expression changes. The most common way to do this is to try to identify the pathway involved, be it either signalling or regulatory. Here, we review three different approaches to the identification and characterization of pathways that contribute to ageing: (i) analysis of protein–protein interactions, (ii) gene-regulatory network analysis, and (iii) modelling quantitative properties of reactions.

#### (i) Protein–protein interaction networks

The analysis of the interplay of proteins mostly begins with collecting a list of proteins, or their respective genes, which have been shown to be involved in ageing. In a second step, the protein–protein interaction (PPI) data are collected, usually from one or more databases such as the Human Protein Reference Database [42] or IntAct [43]. These PPIs define an interaction network, where each protein is a node and each interaction an edge. The network contains the ageing-associated proteins as well as their non-ageing-associated interaction partners.

New ageing-associated genes can then be suggested using a guilt-by-association methodology, i.e. proteins that interact with known modulators of ageing are more likely to regulate ageing than unrelated proteins [44]. This suggestion was also made in a later paper [45], in which the authors showed that the directly interacting proteins are significantly enriched in genes which change their expression during ageing. Additionally, the authors tested 18 directly interacting proteins in *C. elegans* for their influence on ageing using RNAi knockdown. They found a higher percentage of lifespan-extending proteins when compared with genome-wide screens.

PPI network analysis also revealed that highly connected proteins are significantly more likely to be associated with ageing [46]. This was confirmed in independent studies in different laboratories for various organisms such as yeast, fly and human [45,47,48]. Furthermore, Promislow [46] showed a significant positive correlation between connectivity and pleiotropy, which connects the findings to Williams' antagonistic pleiotropy theory of the evolution of ageing [49]. This theory argues that mutations causing ageing can be selected for when they produce beneficial effects earlier in life. However, this does not necessarily explain the high conservation of ageing genes across large evolutionary distances, since different lifestyles can favour different effects. One explanation for this is that ‘what is actively conserved is not the effect of a gene on ageing but the central role of hub genes in biological systems’ [47].

#### (ii) Gene-regulatory networks

A different way to use pathway information is the description of regulatory interactions by ordinary differential equations (ODEs), which describe changes in quantities, in this case changes in gene expressions. This approach has been applied by Lorenz *et al.* [50] to the AMP kinase Snf1 pathway in yeast. The pathway consists of 10 genes and is involved in the caloric restriction paradigm of ageing. The regulations predicted from the ODE model were all checked through gene deletions and chromatin immunoprecipitation quantitative polymerase chain reaction. The final model, although far from perfect, correctly predicted some unknown interactions and suggested biologically meaningful and testable hypotheses [50]. However, it has to be examined how this methodology scales with larger numbers of genes and whether it works in higher organisms. Nevertheless, it is a very interesting study, worth following up.

#### (iii) Quantitative reaction modelling

As more insight is gained on the biological pathways, more detailed analysis of the constituent reactions is possible. Physical properties of pathways are modelled quantitatively, generally by using stochastic simulations. Hucka *et al.* [51] developed the first version of Systems Biology Mark-Up Language (SBML), which attempted to formalize the way biological models and processes can be represented. This is an XML-based, computer-readable format that allows the representation of metabolic and signalling pathways among other biological processes. SBML is extended by Mathematical Mark-up Language (MathML), thus allowing the inclusion of mathematical expressions and the representation of quantitative relations within processes.

Such approaches are just emerging in many aspects of biology and are expected to become increasingly important for understanding complex phenomena such as ageing. Preliminary work on a computational platform that enables the design and execution of quantitative studies has been conducted by Chen *et al.* [52]. Although generally applicable, the tools have been developed with the view of inferring parameters of stochastic models of ageing.

A recent example of the use of SBML for quantitative modelling of ageing processes can be found in a paper by McAuley *et al.* [53], where the authors construct an SBML model to study the chronic effects of elevated plasma cortisol on hippocampal activity and atrophy. The SBML model enabled the authors to integrate a flow diagram representing the structure of the physiological system with the physiological variables using relevant mathematical equations that model enzyme–substrate reactions and receptor–ligand interactions. Their results suggest that acute and chronic increases in the secretion of cortisol induced an age-related hippocampal atrophy and a loss of hippocampus activity. They suggest clinical studies that could be performed in order to investigate whether these *in silico* results are reflecting a real biological effect.

### (d) Image analysis

In the search for markers of ageing, various laboratories have explored not only expression but also automated analysis of microscopy images. The Wolkow and Goldberg laboratories developed a software to automatically estimate the age of *C. elegans* from microscopy images of the terminal bulb. In a first approach, images were described with a high number of features (1025) from which 28 were selected for the final model [54]. In a second approach, they used image texture analysis with two characteristics reflecting grey-level co-occurrence and directionality [55]. In both studies moderate correlations of the prediction with chronological age were found. These methods, fitted to estimate the age of normal living worms from images, can be used to estimate how much younger a long-lived worm looks when compared with its chronological age.

## 4. Comparative analyses of species and tissues

Comparative studies are helpful in identifying the links between the ageing phenotypes and their causes at the molecular level. The data used in comparative studies include information available on ageing phenotypes, experimental datasets and other background knowledge on the organisms' genome and pathways. Apart from developing ways to combine the different data types, comparative studies are also used to investigate the different ageing theories and understand the differences in lifespans between species. Below, we review examples of various comparative analyses.

Research in ageing has predominantly been driven by experiments in yeast and invertebrates, with the fruitfly and the worm being the most widely used ones. These animals are being used so extensively because they have short lifespans and are established laboratory model organisms, which means that their genomes have been sequenced and are relatively well annotated. Mice, with a longer lifespan than worms and flies but shorter evolutionary distance to humans, are also used in many ageing experiments [15,23,56,57] to understand similarities and differences on ageing in the different model organisms. Experimental datasets compare organisms with different longevity phenotypes with the aim of inferring associations or causal relationships between ageing phenotypes and genes, proteins or pathways.

### (a) Within species: comparing genotypes

**Figure 4.**
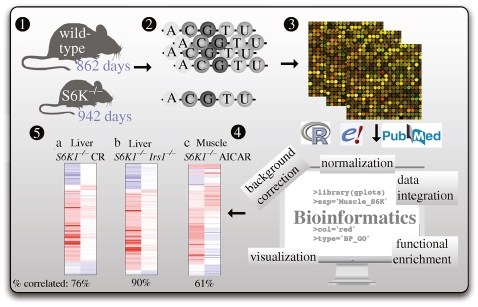
Gene-expression analysis of a longevity experiment. Schematic representation of a standard bioinformatics analysis of an ageing experiment involving microarray data. Selman *et al*. [23] found that global deletion of the *S6K* gene extends lifespan in the mouse. Median lifespan in *S6K1*^−/−^ mice increased by 80 days (from 862 to 942 days) relative to that of wild-type mice (1). After mRNA extraction (2) and hybridization to microarrays (3) bioinformatics methods (4) were applied to establish gene-expression changes between wild-type and mutant mice. Similarities and differences between expression profiles observed for various body parts or associated with other longevity experiments were established (5). Typical steps of such a bioinformatic analysis involve data integration, normalization, background correction, functional analysis and visualization steps. Step 5 shows a comparison of the *S6K1*^−/−^ mice with mice that have been calorie-restricted (CR), that lack the insulin receptor substrate protein 1 (Irs1^−/−^) or that have been treated with the AMPK activator aminoimidazole carboxamide ribonucleotide.

Comparative studies between different genotypes within a species are employed to identify associations between certain genes and ageing. For instance, Selman *et al.* [23] established that mice in which the gene S6 Kinase 1 (*S6K1*) was inactivated live longer and show improved health in later life than control mice. *S6K1* is a ribosomal S6 kinase, a component of the nutrient-responsive mammalian target of rapamycin (mTOR) signalling pathway. Microarray data analysis helped to pinpoint the gene-regulatory changes accompanying the long-lived animals compared with wild-type mice (figure 4). For example, increased expression of genes associated with pathways previously associated with ageing and with caloric restriction, was observed in liver (*Ppargc1a*, *Ppargc1b*, *Foxo1*, *Foxo3a*, *Cpt1b*, *Pdk4*, *Glut1* and *Cyc*) and muscle (*Ppargc1a*, *Ppara*, *Foxo1*, *Foxo3a*, *Pdk4*, *Glut1*, *Sirt1* and *Ucp3*) of *S6K1*^−/−^ mice. Computational analysis of the data also showed that the *S6K1* mice resemble mice that have been treated with AMPK-activating drugs. These results suggest that therapeutic manipulation of *S6K1* and AMPK might mimic caloric restriction and could provide protection against diseases of ageing.

### (b) Within species: tissue specificity

Many ageing-related changes can be associated with changes in tissue-specific gene expression, while there also may be a common set of genes that change equivalently in different tissues [21,58–61]. For example, genes that make up the mitochondrial electron transport chain appear to decrease in expression with age in multiple tissues [62]. In contrast, the effects of ageing are particularly pronounced in the brain, where a reduction in synaptic density has been observed in various organisms [21,63].

To examine transcriptional alterations associated with ageing in various tissues and tissue parts, DNA microarrays have been used to compare the gene-expression profile of various tissues within an organism employing several statistical techniques. For instance, the AGEMAP project [15] investigated the expression changes for 8932 genes in 16 mouse tissues as a function of age. The authors used a multiple regression model to measure changes in expression with age for each of the individual tissues. They found that some tissues displayed large transcriptional differences in old mice, while other tissues showed few or no changes in expression with age. The tissues could be classified into an age-related transcriptional pattern common to neural tissues, a pattern for vascular tissues, and a pattern for steroid-responsive tissues. The authors observed that different tissues age in a coordinated fashion in individual mice, such that certain mice exhibit rapid ageing, whereas others exhibit slow ageing for multiple tissues.

A study in human investigated similarities and differences in expression changes in the cortex and the medulla of the kidney with age [60]. A linear regression model was used to identify genes that showed a statistically significant change in expression with age. The age-regulated genes showed a similar ageing profile in both kidney parts, suggesting a common underlying mechanism for ageing in this organ.

A fruitfly study involving seven tissues at six adult life stages revealed tissue-specific patterns of gene expression [61]. The seven tissues for which genome-wide expression profiles were measured were the brain, thoracic muscle, gut, malpighian tubule, accessory gland, testis and abdominal adipose tissue. In each tissue hundreds of age-related differentially expressed genes were found. Less than 10 per cent of them in each tissue were common with any other tissue. Similarly, less than 20 per cent of the biological processes enriched with the age-related genes were common between any two tissues.

Because of the small size of the fruitfly and worm and the difficulties of dissecting specific tissues for analysis, expression-profiling is still often applied to RNA extracted from whole organisms or from body parts of heterogeneous tissue composition [21,64–66]. While it is known that a significant fraction of the genome will be missed or under-represented in whole animal samples and that tissue-specific expression may be inadequately captured, there is little information concerning the capacity of microarrays to capture tissue-specific effects of ageing, and other biological processes, in whole animal samples. Databases that provide tissue-specific expression profiles can assist in analysing whole animal data. The FlyAtlas database [67] currently provides gene-expression profiles for 17 adult fruitfly tissues. The data can in principle be used for various purposes, for example to investigate the tissue specificity of age-associated changes in whole-animal data via virtual dissection of whole-fly gene-expression data or for the general investigation of tissue specificity of age-associated genes in fruitfly studies.

### (c) Within species: population studies

In addition to molecular studies, population-based genetic studies are becoming increasingly powerful. For example, various datasets have been collected from centenarians and families with members that show exceptional longevity. Certain populations have been identified as long-lived and have now been established as ‘model’ populations for human population studies in longevity. Two of the most studied ones are the Ashkenazi Jews and the Okinawa Centenarians in Japan. Comparisons between these groups and individuals with shorter lifespans can reveal determinants of ageing, such as genes [68], environmental factors [69] or SNPs [70].

The Okinawa Centenarian Study (http://www.okicent.org) focuses on a Japanese sub-population in the area of Okinawa that has a detailed birth and death registry and high incidence of centenarians. This study has produced valuable insight on the role of the environment and diet in longevity and has helped identify physiological biomarkers of human ageing [69]. Using data on lifestyle and caloric intake, together with classical statistics analysis, this study has characterized more thoroughly the clinical features of Okinawan individuals that are associated with their low caloric intake, longer lifespans and healthier ageing, low incidence of diabetes, cancer and cardiovascular disease.

In a study by Bergman *et al.* [68], different groups of humans between the sixth and eleventh decades of their lives were compared. The sample population was taken from a group of long-lived Ashkenazi Jews and their offspring. The authors tested their hypothesis that some of the favourable genotypes in long-lived humans act as mechanisms that buffer the deleterious effect of age-related disease genes. They modelled the results from high-throughput genotyping datasets from the different age groups using a binomial model and identified three candidate gene loci: *apoC-3*, *lpa* and *cetp*. Finally, they constructed pathways between these candidate loci from PPI networks to identify possible mechanisms of their function. These pathways implicated additional genes: *apoE* (apolipoprotein E), *lpal2* (LPA-like lipoprotein), *pltp* (phospholipid transfer protein) and *apoA1* (apolipoprotein A1), thus suggesting candidates for further investigations.

Recently, a Bayesian classification model was developed that predicts exceptional longevity in humans [71]. The model discriminates individuals with exceptional longevity from individuals with average longevity based on 150 SNPs indicative of longevity. When tested on 254 centenarians and 341 controls, the model correctly assigned the group in 77 per cent of the test cases. Closer analysis of individual cases revealed that exceptional longevity is determined by varying combinations of SNPs. The authors used a Bayesian model-based clustering procedure to group the individuals according to their genetic risk profiles related with a varying number of SNPs. They obtained 19 groups of greater than or equal to eight centenarians with similar genetic-risk profiles. The different signatures correlated with differences in the prevalence and age-of-onset of age-associated diseases. The model that was trained on 801 positive and 926 negative examples will have to be confirmed by future studies with a larger number of individuals.

### (d) Between species: evolutionarily conserved ageing processes

Most experimental hypotheses on ageing, especially those involving gene perturbations or environmental stresses, are tested on model organisms. It is useful to be able to extrapolate from those results to other organisms, such as humans, where full experiments are not feasible. Comparative computational analyses between species generally take advantage of the established orthologous relationships between genes of different taxa, in order to find similarities and differences between the ageing processes, filter complex genomics datasets to identify candidate genes or extrapolate from the results on model organisms in order to find candidate ageing genes or processes in others.

**Figure 5.**
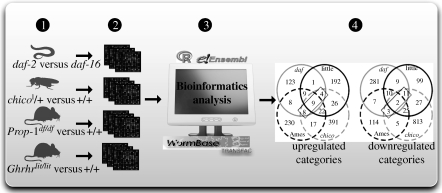
Comparative genomics analysis between species. Schematic representation of a comparative genomics analysis between species, as applied to experiments on ageing. McElwee *et al*. [22] found that there is little evolutionary conservation at the level of individual genes (orthologous or paralogous) under the regulation of the IIS, but there is significant enrichment for genes in some functional categories whose expression changes in long-living animals across species. They first compared the *chico* mutant on the fly against the wild-type and identified the differentially expressed genes from microarray experiments (1,2). Then, they employed publicly available datasets on similar assays in the worm and mice (1,2). Using the orthologous genes between the three animals available in Ensembl, they estimated the statistical significance of the differentially expressed orthologues (3). Finally, they identified the gene categories that are significantly enriched for differentially expressed genes and identified their overlaps in four datasets (4).

For example, McElwee *et al.* [22] focused their comparisons on specific mutants of mice, flies and worms, which affect genes that regulate the IIS pathway. Figure 5 demonstrates their approach. They produced expression datasets and analysed them together with publicly available ones to find differentially expressed genes. Using orthology relationships, they statistically determined the significance of the differentially expressed orthologues, concluding that although there is no significant conservation at the gene level, there is significant conservation at the level of molecular processes. Using the GO database, they identified a group of processes as evolutionarily conserved, including sugar catabolism, energy generation, glutathionine-*S*-transferases and other processes linked to cellular detoxification.

Smith *et al.* [29] used similar methods to identify members of the TOR pathway in yeast and *C. elegans*. McCarroll *et al.* [72] likewise compared experiments in humans, yeast, fly and worm, implicating mitochondrial metabolism, DNA repair and cellular transport as evolutionarily conserved processes involved in ageing.

In an attempt to identify age-related gene-expression signatures that are shared between rats, mice and humans, de Magalhães *et al.* [30] performed a meta-analysis of publicly available age-related expression microarray datasets on healthy, non-treated adult specimens. They first determined the representation of GO categories using standard functional enrichment analysis from the individual microarray experiments. Then, they identified those genes, whose expression is associated with age by linear regression and identified consistently under- or over-expressed genes by employing the cumulative binomial distribution. Using homologous relationships for the cross-species comparisons of their results, they identified the GO categories that are significantly over-represented by ageing-related genes across species. These include inflammation and immune response, energy metabolism, as well as apoptosis, cell cycle and cellular senescence.

## 5. Computational aspects of other ageing studies

Within or between species, studies on ageing aim to investigate and better characterize the different processes of ageing, as well as those diseases that are linked with ageing phenotypes. This section reviews some recent results of studies on the role of DR and oxidative stress as environmental factors influencing ageing and describes some research highlights on age-related diseases. We emphasize the bioinformatics methods used to interpret the data generated by these studies.

### (a) Dietary restriction

Survival analyses on different organisms with DR when compared with fully fed organisms have shown that DR extends lifespan in several organisms [73,74]. Transcriptomic analyses on DR animals have helped to identify genes and processes that are affected by DR and are implicated in the longevity phenotype. For example, a study by Selman *et al.* [75] has analysed whole-genome RNA profiling datasets from liver, skeletal muscle, colon and hypothalamus of acute dietary restricted mice. They identified different processes that are up- or downregulated in the various tissues and they looked at these results in conjunction with plasma metabonomic profiles to conclude that in liver and muscle energetically expensive biosynthetic processes are downregulated, whereas fatty acid metabolism and gluconeogenesis are increased. In muscle and colon they observed that genes involved in cellular proliferation are turned down.

In a different gene-expression microarray study, Tsuchiya *et al.* [76] explored the effects of disrupted growth hormone/insulin-like growth factor-1 signalling and DR in rodent livers. Their computational analysis involved high-throughput gene-expression analysis followed by ANOVA, in order to identify the cause of the changes in gene expression, as being DR, disrupted growth hormone/insulin-like growth factor-1 signalling or both. They identified various categories of genes that are influenced by both factors in an additive way, including gluconeogenesis, apoptosis, xenobiotic and oxidant metabolism that result in a narrowed-down group of genes associated with regulating longevity.

Spindler & Mote [77] investigated the transcriptional effects of glucoregulatory drugs via microarray profiling and compared them with the effects of hepatic long-term and short-term DR. They identified metformin as a drug that produces a similar expression spectrum to DR mice and argued that such comparisons of profiling data could be superior to lifespan studies when screening for longevity drugs.

The relationship between lifespan extension effects of DR and the IIS and TOR pathways is unclear. Most studies on these pathways involve mutants on genes that are important elements of these pathways and compare the mutant organisms to the wild-type ones for lifespan extension and differences in gene expression without investigating the effects on DR [78].

Ageing studies on DR in humans are difficult to perform because of the time scale of human lifespan. There are, however, limited studies on the effects of DR in humans [79]. There have also been studies that monitor surrogate indicators of secondary effects of ageing, such as atherosclerosis and incidence of malignancies. Holloszy & Fontana [80] review a collection of studies on the effects of 3–14 years of DR in a group of humans, and show that these individuals have lower levels of atherosclerosis, less incidence of diabetes or malignancies. The data in all these studies are compared using classical statistical methods.

Recently, a survival analysis of DR rhesus monkeys was published by Colman *et al.* [81]. The study showed that lifespan is extended in the DR monkeys and the onset of age-related phenotypes is delayed. A previous study by Bodkin *et al.* [82] had also shown an increase in the average age of rhesus monkeys, prevention of hyperinsulinaemia and the alleviation of age-related disease by DR. This is an important result, as it provides greater evidence that the effects of DR on lifespan are conserved in higher mammals and in particular primates.

### (b) Oxidative stress

One of the theories of ageing predicts that interventions changing the levels of oxidative stress alter ageing [83]. Therefore, a number of comparative studies aim to understand how oxidative stress can regulate lifespan. For example, Curtis *et al.* [84] investigated the transcriptional effects of over-expressing manganese superoxide dismutase (MnSOD) and the resulting increase in lifespan in the fly. They first observed a decrease in metabolic rate of the flies. Through transcriptional profiling they identified candidate biomarkers for ageing that are enriched for electron transport and carbohydrate metabolism genes. Finally, they compared the fly datasets to the genes regulated by insulin-like signalling in long-lived worm mutants to identify common ageing genes between the two species. Their computational analysis identified genes involved in various processes, such as the purine biosynthetic pathway, programmed cell death and insulin signalling.

On the other hand, there has been no evidence that over-expressing superoxide dismutases can increase lifespan in mice and worms [85,86]. Comparisons between species with different rates of ageing have been informative in finding out the relationship between oxidative stress and ageing. For example, Salmon *et al.* [87] compared the protein oxidation levels of species of long-lived bats to short-lived mice using statistical analyses and found that the bat species had a significantly lower protein oxidation. These findings suggest, but do not prove, that long-lived species might have reduced oxidative damage. In a different comparative study, Mitchell *et al.* [88] compared the composition of membrane phospholipids in long-lived mole rats to short-lived mice as an indication of antioxidant defences. They used mass-spectrometry shotgun lipidomics and analysed the results statistically to identify and quantify the phospholipids in each of the two organisms. The composition of phospholipids in mole rats suggested that these animals are more resistant to peroxidative damage than short-lived mice.

### (c) Diseases and ageing

The relationship between diseases and ageing has also been researched. A computational study by Wang *et al.* [89] revealed an overlap of disease genes with ageing genes in human which is more than three times higher than expected by chance. Additionally, a grouping into ageing-related diseases and non-related ones was created by means of the overlap of ageing genes with the respective disease genes. This grouping shows further discriminating properties such as the percentage of essential genes, GO annotations, as well as the ratio of non-synonymous to synonymous or silent mutations [89]. The authors described these by constructing a network of the ageing and disease genes, using PPI network information and performed topological analyses to describe its features.

The study on exceptional longevity in humans mentioned above [71] did not observe a substantial difference in the numbers of disease-associated variants carried by centenarians and controls. The authors suggested that centenarians do not lack disease-associated variants but counter the effect of disease-risk alleles.

Cancer, as a disease most prevalent in old age, has been associated with ageing. Budovsky *et al.* [90] analysed more thoroughly the relationship between longevity and cancer-related genes and proteins. They first investigated the evolutionary conservation of longevity related genes from model animals to humans using orthology relations. Similarly, they also found conserved human cancer-related genes in model organisms. They observed a significant overlap between those two groups of genes. They further characterized these genes and found that genes orthologous to human tumour suppressors dominate among lifespan-increasing genes in all species. In addition, genes orthologous to human oncogenes dominate among the lifespan-reducing genes. After pathway analyses, they discovered that these common proteins tend to be highly connected (hubs) in the PPI network and that the majority are involved in signalling and transcription regulation.

Machine learning methods have been used to detect early stages of Alzheimer disease (AD) [91]. In this study, deformation patterns of hippocampus between patients with AD and healthy control subjects could be detected. Hippocampus images were acquired from 19 patients with AD and 20 control subjects. Regional changes of bilateral hippocampi were characterized using computational anatomic mapping methods. Patients with AD showed significant deformations in a region of bilateral hippocampi. The accuracy of the classification algorithm was given as 80 per cent.

## 6. Conclusion

Even though the data originating from diverse ageing studies require different methods for their interpretation, they have one thing in common, namely that they rely on bioinformatics methods. Bioinformatics is ubiquitous in the sense that it is widely used in all fields of ageing studies, ranging from demographical to genomic and proteomic studies.

Text-mining, although still in its infancy, could prove to be a useful tool for ageing research with to date nearly 240 000 articles found in PubMed when searching for ‘ageing’. We have seen that bioinformatics is essential from data handling to interpretation and integration to complex comparisons, hypotheses and model building. The majority of experimental approaches in the field of ageing involve functional genomics experiments, such as high-throughput expression profiling and sequencing. Integrating these resulting datasets with data from functional biochemical experiments could lead to more informative results on the mechanisms of ageing. As we gain more knowledge of complex ageing processes, the need for better modelling tools is becoming even more critical, placing computational biology at the heart of ageing research in the future.
